# Stroke specific quality of life questionnaire: Test of reliability and validity of the Persian version

**Published:** 2015-04-04

**Authors:** Mojtaba Mahmoodi, Anahid Safari, Mehrdad Vossoughi, Fatemeh Golbon-Haghighi, Maliheh Kamali-Sarvestani, Haleh Ghaem, Afshin Borhani-Haghighi

**Affiliations:** 1 Health Policy Research Center AND School of Medicine, Shiraz University of Medical Sciences, Shiraz, Iran; 2 Department of Pharmacology, School of Medicine, Islamic Azad University, Kazeroon Branch, Kazeroon, Iran; 3 Department of Dental Public Health, School of Dentistry, Shiraz University of Medical Sciences, Shiraz, Iran; 4 Student Research Committee, Shiraz University of Medical Sciences, Shiraz, Iran; 5 Department of Epidemiology, School of Health, Shiraz University of Medical Sciences, Shiraz, Iran; 6 Clinical Neurology Research Center AND Department of Neurology, Shiraz University of Medical Sciences, Shiraz, Iran

**Keywords:** Stroke, Quality of Life, Reproducibility of Results, Questionnaires

## Abstract

**Background: **The aim was to assess the reliability and the validity of the translated version of the stroke specific quality of life (SS-QOL) questionnaire in Iranian post-stroke patients.

**Methods:** This project was performed at the Shiraz University of Medical Sciences, Shiraz, Iran, between 12 April 2010 and 24 February 2011. The English version of the SS-QOL was translated into Persian by “forward-backward” translation, cognitive inquiring and cultural adaptation process. The reliability and internal consistency were measured by Cronbach’s alpha coefficient. Validity was assessed using convergent and divergent validity through Spearman’s correlation coefficient.

**Results: **Our study included 117 post-stroke patients, consisting of 57 (48.7%) men and 60 (51.3%) women. The mean age of the patients was 81.60 ± 7.52 (range 60-88) years. The Persian version of the SS-QOL proved reliable (Cronbach’s α = 0.96). Internal consistency was excellent for both demographic and patients’ clinical characteristics (Cronbach’s α ≥ 0.70). The scaling success rates were 100% for convergent validity of each scale. Divergent validity for all 12 scales was considered acceptable, whereas each scale had a 100% scaling success rate for convergent validity.

**Conclusion: **The Persian version of SS-QOL should be mentioned as a noteworthy instrument to specify different aspects of health related QOL of patients suffering stroke and hence that clinicians, researchers and epidemiologist can exploit it trustfully.

## Introduction

Stroke is the foremost cause of adult disability worldwide. Although the stroke considered as the third cause of mortality in developed countries,^1^ it is ranked as the second cause of death in developing counties.^2^ The socioeconomic importance of this non-communicable disease is growing in ageing populations.^3^ It also represents a major cause of long-term disability with a potentially major impact on patients, their families, and health-care services by various emotional and socioeconomic aspects.^4^ Stroke mortality data from multiple countries reveal that, as a whole, mortality rates have decreased in recent decades.^5^^,^^6^ Stroke incidence, as first ever event of its kind, was estimated between 22.7 and 103.23/1,00,000 individuals in all age ranges. These statistics showed that approximately 70% of the patients survived the acute initial phase. The increasing number of long-term post-stroke survivors due to improved medical and social care, successful and effective secondary prevention especially by antihypertensive agents and incremental overall life expectancy demonstrate the unique and specific role of stroke in the drafting and implementation of healthcare strategies.^7^^,^^8^

The majority of the surveys evaluating the quality of life (QOL) after stroke have applied generic instruments such as the Short Form-36, the well-being scale, the sickness impact profile, the EuroQOL, or the Nottingham Health Profile. These scales enable researchers compare patients with different diseases, but are less sensitive regarding the specific effects of a specific disease, such as stroke, on the patient’s QOL or the response to a specific treatment.^9^ The stroke specific QOL (SS-QOL) questionnaire is one of the noteworthy specific scales for the determination of QOL after stroke, which is significantly more valid and sensitive as compared to traditional instruments.

Currently, there are approximately more than 80 million persons who speak Persian worldwide. They live in Iran, Afghanistan, Tajikistan, Uzbekistan and several other countries. There has been no valid and reliable questionnaire with official Persian language to evaluate QOL in stroke patients in Iran. The current study was conducted in order to translate and validate the Persian version of the SS-QOL.

## Materials and Methods

The SS-QOL, which is a disease-specific QOL measure, consists of 49 items encompassing 12 domains, which include the social role (five questions), mobility (six questions), energy (three questions), language (five questions), self-care (five questions), mood (five questions), personality (three questions), thinking (three questions), upper extremity function (five questions), family role (three questions), vision (three questions), and work/productivity (three questions). Each item is ranked on a five-point Likert scale in which level one means completely agreed while level five means completely disagree. The summary score of this scale is an un-weighted average of the 12 domains. The total score ranges from 49 to 245, with higher scores indicating a better QOL.

This face-to-face interview survey was performed at the Stroke Special Clinic, Department of Neurology, Shiraz University of Medical Sciences, Shiraz, Iran between 12 April 2010 and 24 February 2011. For translating the questionnaire from English to Persian, the standard forward-backward method was used, as described by our previous studies.^10^^,^^11^ All 49 items were translated by expert bilinguals into Persian and afterwards, the preliminary version was again translated into English. Cultural adaptation was performed in order to obtain a version of the questionnaire that is practically as similar as possible to the main English one, along with patients’ perception and understanding. The Persian version of SS-QOL was filled out by 20 patients. All these patients were asked to evaluate the transparency and clarity of each question. All the findings of this pilot study and the interviews with the patients were gathered. Based on the results of this pilot study, unclear or questionable items were modified.

In general, the patients reported that they had no problems in understanding and answering all of the questions of the Persian version of SS-QOL.

All demographic data including age, sex, marital status, dwelling place, educational and socioeconomic status were registered. A qualified neurologist was responsible for gathering clinical and medical data of patients related to stroke, comprising of the type of stroke, duration of disease, etc.

Patient inclusion criteria were age above 50 years and proved diagnosis of stroke. Stroke was defined according to National Clinical Guideline for Diagnosis and Initial Management of Acute Stroke and Transient Ischemic Attack (TIA)^12^ and diagnosis was confirmed by clinical history, neurological examination and imaging via computed tomography scan and/or magnetic resonance imaging. The sample was selected from literate and illiterate people who accepted to participate in this study. Patients with any known vasculitis, thrombophilic diseases, infectious vasculopathy, arterial dissection, moyamoya disease, radiation induced vasculopathy, fibromuscular dysplasia, sickle cell disease, neurofibromatosis, reversible cerebral vasoconstriction syndrome, vasospasm after subarachnoid hemorrhage and cerebral venous sinus thrombosis were excluded from the study. Those with TIA without progression to stroke as well as those with severe heart, liver or renal disease that may considerably influence the QOL were also excluded.

Individuals were interviewed personally by family physicians under the full observation of a neurologist. The questions were asked during a face-to-face interview in Persian. The interviewer intervened only to clarify a question if required, but did not reveal any information about the value of each item or effect of each question on the outcome. No attempt was made to prompt the respondents by suggesting answers directly.

The questionnaire was filled out by literate subjects. For illiterate subjects, the questions were asked through an interview in Persian. The interviewer could only explain the meaning of questions for illiterate patients. The relevance and clarity of the questions were also assessed.

The approvals of the Institutional Review Board, as well as the Ethics Committee of the Shiraz University of Medical Sciences, were obtained before the start of the study. All participants gave their written informed consents. This study was designed and performed according to principles of Helsinki Declaration.

Previous studies recommend that the acceptable sample size for testing the validity and reliability of QOL questionnaires are between 100 and 400.^13^

Statistical analyses were performed using the SPSS software, (version 17.0, SPSS Inc., Chicago, IL, USA). Results are reported as the mean ± standard deviation or n (%), as appropriate. The SS-QOL scale scores were measured using the Likert method for summed ratings, and the raw scores were linearly transformed into 49-245 scales: the higher the transformed score, the better the patient’s health related QOL (HR-QOL).

The internal consistency and reliability were examined using Cronbach’s alpha (recommended value α ≥ 0.70).^13^^-^^15^ To assess the validity (convergence and divergence) of SS-QOL questionnaire, the Spearman's correlation coefficient was used.^14^^,^^15^ Convergence validity assesses the relevance of each item with the subscale containing it. Divergence validity assesses the irrelevance of each item with the subscales not containing it. It is generally expected that an item has a high correlation with its subscale and low correlation with other subscales.

To determine the psychometric properties of the questionnaire’s scales for ceiling effect, we counted the percentage of subjects who scored five for each item, and to determine the floor effect, we counted the percentage of subjects who scored one for each item.

## Results

Overall, we included 117 post-stroke patients among whom there were 57 (48.7%) men and 60 (51.3%) women. The mean age of the patients was found to be 81.60 ± 7.52 (range 60-88) years. The majority of patients (82.1%) were married. Duration of the disease was 1.92 ± 1.86 years (range 0.8-12). 54 (47.8%) patients were illiterate, whereas 41 (36.3%) patients were semi-literate. Both high school graduates and university degrees included 9 (8%) patients. A total of 108 (92.3%) patients suffered ischemic stroke while the rest, 9 (7.7%) patients, suffered a hemorrhagic stroke (Table 1).

The reliability of the whole 49 questions was provided by the Cronbach’s alpha coefficient (α = 0.96), whereas the individual coefficients according to sex, marital status, residency, education, stroke type and duration of disease are shown in table 2.

Based on the correlation coefficients, there is an acceptable association between each scale and its items (recommended r ≥ 0.40) while the scaling success rates were 100% for the convergent validity of each scale. Internal consistency for all scales, except for overall QOL is excellent (α ≥ 0.70; range: 0.74-0.94). On the other hand, each scale shows the least associations with other items in discriminate scales. Therefore, divergent validity for all scales (regarding corresponding discriminate scales) is satisfactory (Table 3).

**Table 1:** Demographic, socioeconomic and clinical characteristics of 117 post-stroke patients

**Variable**	**n (%)**	**Mean ± SD**
Sex		-
Male	57 (48.7)	-
Female	60 (51.3)	-
Marital status		-
Single	21 (17.9)	-
Married	96 (82.1)	-
Educational status		-
Illiterate	54 (46.2)	-
Semi-literate	41 (35.0)	-
High school	9 (7.7)	-
University degree	9 (7.7)	-
Residency		-
Urban	87 (74.4)	-
Rural	30 (25.6)	-
Type of stroke		-
Ischemic	108 (92.3)	-
Hemorrhagic	9 (7.7)	-
Age (Range)		81.60 ± 7.52 (60-88)
Disease duration (years) (Range)		1.92 ± 1.86 (0.8-12)
Disease severity (BI score) (Range)		79.00 ± 22.61 (20-105)

SD: Standard deviation; BI: Barthel Index

**Table 2:** Internal consistency of Stroke specific quality of life (SS-QOL) questionnaire in different demographic, socioeconomic and clinical subgroups

**Variable**	**Number**	**Cronbach’s coefficient**
Age		
< 70	14	0.96
70-85	42	0.96
≥ 85	61	0.97
Sex		
Male	57	0.97
Female	60	0.95
Marital status		
Single	21	0.96
Married	96	0.96
Educational status		
Illiterate	54	0.96
Semi-literate	41	0.95
High school	9	0.89
University degree	9	0.98
Residency		
Urban	87	0.97
Rural	30	0.96
Type of stroke		
Ischemic	108	0.97
Hemorrhagic	9	0.96
Disease duration (year)		
< 1	45	0.97
1-3	36	0.96
≥ 3	36	0.95
Disease severity (BI score)		
Mild	71	0.94
Moderate	28	0.88
Severe	18	0.87

BI: Barthel index

Interestingly, the values of the Cronbach’s alpha coefficients are all excellent in the subgroups according to gender and marital status (α ≥ 0.70; range: 0.70-0.95), except for that of family role subscale for singles (α = 0.39) (Table 4).

Concerning the internal consistency of each subscale of SS-QOL regarding stroke type and disease, the α values are interestingly excellent, with only one exception in the case of language subscale for patients with hemorrhagic stroke (α = 0.33) (Table 5).

Table 6 shows the floor and ceiling effects for each subscale of the questionnaire. The ceiling effects were generally greater than floor effects. However, the amount of values indicated that the variability to the subscales were generally acceptable and have not been affected by the accumulation of same responses in a specific item. 

## Discussion

The efficacy of interventions in stroke has been evaluated mainly on the basis of clinical endpoints, although patients and their families face a range of psychosocial issues. As a consequence, a variety of stroke-specific questionnaires has been developed for the assessment of HR-QOL,^16^ an important concept to better understand the distress of people with stroke. Nowadays, it is widely used as a HR-QOL indicator for post-stroke individuals. It has been validated for use in Croatia,^17^ Malaysia,^18^ Taiwan,^19^ the Netherland,^20^ Brazil,^21^ Denmark,^22^ Germany,^23^ Great Britain,^24^ the United States.^25^

**Table 3:** Convergent validity for stroke specific quality of life (SS-QOL); Item scaling tests

**Scale**	**Number of items per scale**	**Convergent validity (range of correlation)**	**Scaling success** ^*^	**Scaling success** ^**^	**Internal consistency (Cronbach’s alpha)**	**Divergent validity (range of correlation)**
Energy	3	0.75-0.89	3/3	100	0.80	0.08-0.49
Family role	3	0.68-0.88	3/3	100	0.74	0.03-0.78
Language	5	0.75-0.83	5/5	100	0.94	0.13-0.47
Self-care	5	0.77-0.91	5/5	100	0.92	0.12-0.82
Social role	5	0.76-0.88	5/5	100	0.88	0.14-0.72
Thinking	3	0.82-0.87	3/3	100	0.81	-0.08-0.45
Mood	5	0.53-0.85	5/5	100	0.80	0.14-0.53
Personality	3	0.85-0.90	3/3	100	0.86	0.14-0.58
Upper extremity function	5	0.79-0.84	5/5	100	0.92	0.11-0.79
Vision	3	0.82-0.86	3/3	100	0.85	0.06-0.37
Work (Productivity)	3	0.81-0.92	3/3	100	0.86	0.11-0.78
Mobility	6	0.79-0.93	6/6	100	0.94	0.05-0.78

Spearman’s correlation coefficient was used for assessing convergent and divergent validities of each scale and corresponding items

* Number of correlations between items and hypothesized scale corrected for overlap > 0.4/total number of convergent validity tests;

** Scaling success rate of previous column as percentage

**Table 4:** Internal consistency (Cronbach’s alpha) of each subscales of stroke specific quality of life (SS-QOL) questionnaire by gender and marital status

**Scale**	**Cronbach’s alpha**			
	**Male**	**Female**	**Single**	**Married**
Energy	0.87	0.87	0.76	0.81
Family role	0.77	0.72	0.39	0.79
Language	0.94	0.93	0.93	0.93
Self-care	0.93	0.92	0.94	0.92
Social role	0.90	0.84	0.85	0.89
Thinking	0.80	0.83	0.90	0.79
Mood	0.82	0.76	0.70	0.82
Personality	0.86	0.85	0.94	0.84
Upper extremity function	0.93	0.91	0.95	0.91
Vision	0.76	0.90	0.75	0.87
Work (productivity)	0.92	0.79	0.86	0.87
Mobility	0.95	0.92	0.95	0.93

**Table 5:** Internal consistency of each subscale of stroke specific quality of life (SS-QOL) questionnaire regarding stroke type and duration of disease expressed in years)

**Scale**	**Cronbach’s alpha**				
	**Ischemic**	**Hemorrhagic**	**Disease duration ** **(< 1)**	**Disease duration ** **(1-3)**	**Disease duration ** **(≥ 3)**
Energy	0.80	0.80	0.80	0.80	0.82
Family role	0.74	0.79	0.79	0.73	0.70
Language	0.94	0.33	0.93	0.95	0.93
Self-care	0.92	0.88	0.95	0.91	0.88
Social role	0.88	0.92	0.92	0.82	0.89
Thinking	0.80	0.92	0.79	0.81	0.84
Mood	0.80	0.77	0.79	0.80	0.84
Personality	0.87	0.72	0.89	0.84	0.83
Upper extremity function	0.92	0.84	0.94	0.91	0.85
Vision	0.84	0.96	0.90	0.81	0.69
Work (productivity)	0.84	0.72	0.85	0.86	0.88
Mobility	0.94	0.89	0.95	0.94	0.92

**Table 6:** The ceiling and floor effect for the subscales of stroke specific quality of life (SS-QOL)  questionnaire

**Subscale**	**Floor effect (%)**	**Ceiling effect (%)**
Energy	23.1	8.5
Family role	20.5	15.4
Language	3.4	34.2
Self-care	1.7	36.8
Social role	12.0	12.8
Thinking	1.7	33.3
Mood	1.7	20.5
Personality	29.9	13.7
Upper extremity	5.2	32.8
Vision	1.7	54.7
Work product	11.1	20.5
Mobility	4.3	16.2

This survey was conducted with the purpose of translating the SS-QOL questionnaire into Persian and to evaluate its reliability and validity among Iranian post-stroke patients. The psychometric characteristics of the Persian adaptation of the SS-QOL questionnaire were highly satisfactory and compatible to those of the Croatian,^17^ Malaysian,^18^ Taiwanian,^19^ Dutch,^20^ Brazilian,^21^ Danish,^22^ German,^23^ and English^24^^,^^25^ versions.

This study investigated the issue of validity specifically based on convergence and divergence of items in the questionnaire. Our confident finding on the internal validity can be followed by the other researchers to assess the external validity of Persian version of the questionnaire.

Cronbach’s alpha coefficient was applied for determining the reliability, and it was excellent (α ≥ 0.70) for all 12 subscales, making our findings compatible with those of a previous study in Denmark.^22^

The internal consistency of the whole 49 items of the SS-QOL was excellent for both literate and illiterate patients. Cronbach’s alpha was excellent regarding age, sex, marital status, residency, educational status, stroke type and duration of disease.

The present study shows a high convergent validity for all subscales of the SS-QOL questionnaire.

In correspondence to a German trial,^23^ the scaling success rate for all subscales was 100% in our study, whereas the divergent validity for all subscales was acceptable. However, it is interesting to note that this finding has not yet been reported by other surveys.

One limitation of our study was that we were unable evaluate reliability through test-retest analysis. This setting claims for further studies in order to examine the test re-test reliability in order to find more reliable results. Another possible limitation of our study is that minority of the respondents were interviewed while others filled the forms by themselves. This was inevitable due to the fact that some of the stroke patients were illiterate and could not fill out the forms by themselves. It might be a source of bias in our tool validity survey.

Currently, to the best of our knowledge, this is the first study to show the reliability and validity of the Persian version of SS-QOL. The results of our survey proved that the Persian version of SS-QOL has an efficiently structured specification and convergent validity. In addition, this instrument can be used for assessing the effects of stroke on the QOL reliably and confidently.

## Conclusion

In conclusion, we may thereby declare to have accomplished the translation, cultural adaptation and testing of reliability and validity of the SS-QOL questionnaire for Iranian patients. Consequently, the Persian version of SS-QOL should be mentioned as a noteworthy instrument to specify different aspects of HR-QOL for patients suffering a stroke and clinicians, researchers and epidemiologist can exploit it trustfully.

## Notes:

**How to cite this article: **Mahmoodi M, Safari A, Vossoughi M, Golbon-Haghighi F, Kamali-Sarvestani M, Ghaem H, et al. Stroke specific quality of life questionnaire: test of reliability and validity of the Persian version. Iran J Neurol 2015; 14(2): 94-100.
